# Macrophage-Derived Human Resistin Is Induced in Multiple Helminth Infections and Promotes Inflammatory Monocytes and Increased Parasite Burden

**DOI:** 10.1371/journal.ppat.1004579

**Published:** 2015-01-08

**Authors:** Jessica C. Jang, Gang Chen, Spencer H. Wang, Mark A. Barnes, Josiah I. Chung, Mali Camberis, Graham Le Gros, Philip J. Cooper, Cathy Steel, Thomas B. Nutman, Mitchell A. Lazar, Meera G. Nair

**Affiliations:** 1 Division of Biomedical Sciences, School of Medicine, University of California Riverside, Riverside, California, United States of America; 2 Malaghan Institute of Medical Research, Wellington, New Zealand; 3 Laboratorio de Investigaciones FEPIS, Quinindé, Ecuador; 4 Centro de Investigación en Enfermedades Infecciosas, Pontificia Universidad Católica del Ecuador, Quito, Ecuador; 5 St George's University of London, London, United Kingdom; 6 Laboratory of Parasitic Diseases, National Institutes of Health, Bethesda, Maryland, United States of America; 7 Institute of Diabetes, Obesity, and Metabolism, Perelman School of Medicine, University of Pennsylvania, Philadelphia, Pennsylvania, United States of America; Washington University, United States of America

## Abstract

Parasitic helminth infections can be associated with lifelong morbidity such as immune-mediated organ failure. A better understanding of the host immune response to helminths could provide new avenues to promote parasite clearance and/or alleviate infection-associated morbidity. Murine resistin-like molecules (RELM) exhibit pleiotropic functions following helminth infection including modulating the host immune response; however, the relevance of human RELM proteins in helminth infection is unknown. To examine the function of human resistin (hResistin), we utilized transgenic mice expressing the human resistin gene (h*Retn*Tg^+^). Following infection with the helminth *Nippostrongylus brasiliensis* (*Nb*), hResistin expression was significantly upregulated in infected tissue. Compared to control h*Retn*Tg^−^ mice, h*Retn*Tg^+^ mice suffered from exacerbated *Nb*-induced inflammation characterized by weight loss and increased infiltration of inflammatory monocytes in the lung, along with elevated *Nb* egg burdens and delayed parasite expulsion. Genome-wide transcriptional profiling of the infected tissue revealed that hResistin promoted expression of proinflammatory cytokines and genes downstream of toll-like receptor signaling. Moreover, hResistin preferentially bound lung monocytes, and exogenous treatment of mice with recombinant hResistin promoted monocyte recruitment and proinflammatory cytokine expression. In human studies, increased serum resistin was associated with higher parasite load in individuals infected with soil-transmitted helminths or filarial nematode *Wuchereria bancrofti*, and was positively correlated with proinflammatory cytokines. Together, these studies identify human resistin as a detrimental factor induced by multiple helminth infections, where it promotes proinflammatory cytokines and impedes parasite clearance. Targeting the resistin/proinflammatory cytokine immune axis may provide new diagnostic or treatment strategies for helminth infection and associated immune-mediated pathology.

## Author Summary

Parasitic helminths, which infect an estimated two billion people worldwide, represent a significant global public health problem. Infection is associated with life-long morbidity including growth retardation and organ failure. Despite these debilitating conditions, there are currently no successful vaccines against helminths. Further, great variability in the host immune response to helminths exists, with the ability of some individuals to develop immunity, while others are susceptible when re-exposed or maintain life-long chronic infections. Identifying new factors that are differentially expressed in immune versus susceptible individuals could provide new targeting strategies for diagnosis or treatment of helminth infection. Here, we identify an important immunoregulatory function for human resistin in helminth infection. Employing transgenic mice in which the human resistin gene was inserted, we show that human resistin is induced by infection with the helminth *Nippostrongylus brasiliensis*, where it promotes excessive inflammation and impedes parasite killing. Moreover, analysis of clinical samples from two cohorts of individuals infected with filarial nematodes or soil-transmitted helminths revealed increased resistin and serum proinflammatory cytokines compared to putatively immune individuals. Together, these studies suggest that human resistin is a detrimental cytokine that is expressed in multiple helminth infections, mediates pathogenic inflammation, and delays parasite clearance.

## Introduction

Helminth infections are a major global health issue, causing severe morbidity and malnutrition in over a billion people worldwide [1]. Infection with lymphatic-dwelling filarial nematodes can result in lymphedema and elephantiasis, while soil-transmitted helminths (STH) can cause anemia, gastrointestinal hemorrhage or blockage, growth retardation and cognitive impairment. In human gastrointestinal helminth infections, although most infections have shown mild alterations to pathology, severe infections can result in colitis and dysentery [2]. Clearance of helminth infection has relied solely on anthelmintic drugs, including diethylcarbamazine, albendazole and ivermectin; however, there is currently no vaccine against human helminth pathogens and the rate of reinfection remains high. In mouse models, the T helper type 2 (Th2) immune response is essential in immunity to helminths, with CD4^+^ T cells, innate lymphoid cells, eosinophils and basophils as the major producers of the Th2 cytokines, IL-4 and IL-13 [3]–[5]. In mice lacking IL-4, IL-13 or their receptors, there is impaired clearance of helminths, including the soil-transmitted helminth *Nippostrongylus brasiliensis* (*Nb*) and filarial nematodes [6], [7]. In addition to mediating immunity to helminths, Th2 cytokines are critically involved in suppressing infection-induced immunopathology. For instance, *Schistosoma mansoni* infection is lethal in IL-4Rα^−/−^ mice and following *Nb* infection, IL-4Rα^−/−^ mice suffer from exacerbated inflammation in the lungs compared to wild-type mice [8], [9].

The balance between Th2 cytokines and type 1 proinflammatory cytokines, such as IFNγ and TNFα, is also an important consideration for the outcome of helminth infection as these cytokine pathways are counter-regulatory. For example, inhibition of IFNγ promotes expulsion of *Trichuris muris* by allowing the development of a protective Th2 immune response [10]. In filarial nematode infection, severe lymphatic pathology is associated with increased IFNγ and TNFα and conversely decreased IL-4 [11]. Through promoting type 1 inflammatory cytokines, Toll-like receptor (TLR) signaling can negatively impact the host immune response to helminths by delaying parasite expulsion and promoting immune-mediated pathology [12], [13]. Identifying factors that regulate the balance between type 1 and type 2 cytokines may offer new immune targeting strategies to promote protective immunity or ameliorate infection-associated pathology.

Resistin is a member of the resistin-like molecule (RELM) family of cysteine-rich secreted proteins that are conserved in humans and mice. In mice, there are four RELM proteins (resistin, RELMα, RELMβ, and RELMγ), however, there are only two RELM proteins in humans (resistin and RELMβ) [14]. Among the murine RELM proteins, RELMα and RELMβ proteins are potently induced following helminth infection. Studies from our lab and others have shown that RELMα suppresses Th2 cytokine responses induced by *S. mansoni* or *Nb* [15], [16]. On the other hand, the function of RELMβ during helminth infection may be dependent on the helminth parasite. For example, RELMβ induced CD4^+^ T cell production of IFNγ but had no effect on worm expulsion during *T. muris* infection [17]. In response to *Nb* or *Heligmosomoides polygyrus* however, RELMβ was an effector molecule that could interact with the parasite and promote expulsion. Other studies have shown that RELMβ can bind to and inhibit the sensory function of *Strongyloides stercoralis* [18]. These studies, however, have only examined murine RELM proteins and to date, none have investigated the role of human RELM proteins such as resistin in helminth infection.

Murine resistin was initially identified as a hormone secreted by adipocytes that caused insulin resistance, a disorder that can result in type 2 diabetes [19]. Interestingly, although human resistin and mouse resistin have high sequence homology, resistin in humans is predominantly expressed by monocytes and macrophages and not adipocytes [20], [21]. In addition to diabetes, human resistin is also elevated in several inflammatory diseases such as atherosclerosis and rheumatoid arthritis [22], [23]. Indeed, in an inflammatory environment promoted by LPS stimulation, human resistin was upregulated and contributed to inflammation and insulin resistance [24]–[26]. Human resistin also mediated lung cancer cell migration, differentiation of osteoclasts [27], and VEGF-mediated angiogenesis [28]. Despite the many studies focusing on resistin in metabolic disorders and cancer, its function during infection is currently unknown. In human studies with filarial-infected individuals, *Brugia malayi* antigen increased resistin mRNA in human monocytes, particularly when these monocytes were isolated from filarial-infected humans [29], prompting our investigation of the function of human resistin in helminth infection.

To examine the role of human resistin in helminth infection, we employed transgenic mice that express human resistin. These mice are deficient in murine resistin, and engineered to include the hResistin gene and its entire regulatory region (h*Retn*Tg^+^) [26]. In the original study, h*Retn*Tg^+^ mice exhibited a similar hResistin expression pattern as that observed in humans. Additionally, h*Retn*Tg^+^ mice suffered from increased insulin resistance following LPS-induced endotoxemia. These results suggested that hResistin might be functional in mice, and that h*Retn*Tg^+^ mice provide a useful tool to study how hResistin expression is regulated and its function *in vivo*. Following *Nb* infection, we show that hResistin is significantly upregulated by macrophages in the infected lungs and intestine of h*Retn*Tg^+^ mice. Compared to littermate control mice that did not express hResistin (h*Retn*Tg^−^), infected h*Retn*Tg^+^ mice suffered from exacerbated infection-induced inflammation, including increased weight loss and elevated monocyte infiltration in the lung. We performed RNAseq analysis to identify differentially expressed genes that may be responsible for hResistin-mediated effects. Gene set enrichment analysis revealed that hResistin induced global gene ontology pathways that were associated with the inflammatory response, chemokine signaling and TLR signaling. Additionally, exogenous treatment of naïve mice with recombinant human resistin could recapitulate the monocyte responses and gene expression profile observed in h*Retn*Tg^+^ mice, confirming that human resistin directly promotes these inflammatory pathways *in vivo*. Functionally, hResistin-mediated stimulation of the inflammatory immune response led to increased parasite egg burdens and delayed worm expulsion. Finally, in human studies, we observed that patients infected with STH or the filarial nematode *Wuchereria bancrofti* had elevated serum resistin, and that resistin was positively correlated with parasite burden and serum proinflammatory cytokines. Taken together, our findings suggest that human resistin expression is an innate response to multiple helminths, where it promotes monocyte recruitment and a type 1 proinflammatory cytokine environment, leading to impaired helminth clearance and exacerbated infection-associated inflammation.

## Results

### hResistin is upregulated in macrophages following *Nippostrongylus brasiliensis* infection

**Figure 1:**
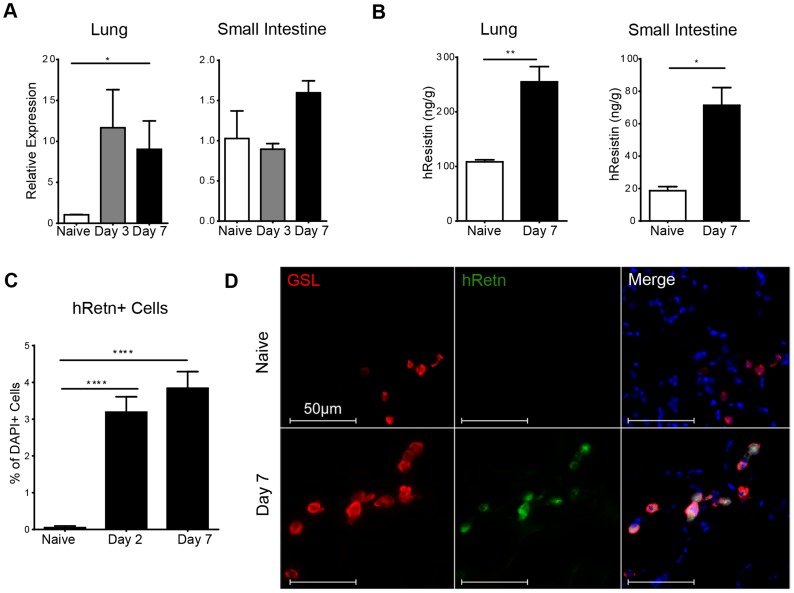
Detection of hResistin expression in *Nb*-infected transgenic mice. (A-B) hResistin expression in the lung and small intestine of naïve or *Nb*-infected mice was measured by real-time PCR analysis (A) or ELISA (B). (C) Quantification of hResistin expressing cells was performed on immunofluorescent stained lung sections. (D) IF staining for *Griffonia simplicifolia* lectin (red), hResistin (green), and DAPI (blue) was performed on lung tissue sections. Data (mean ± SEM, n = 4–6 per group) are representative of four separate experiments.

**Figure 2:**
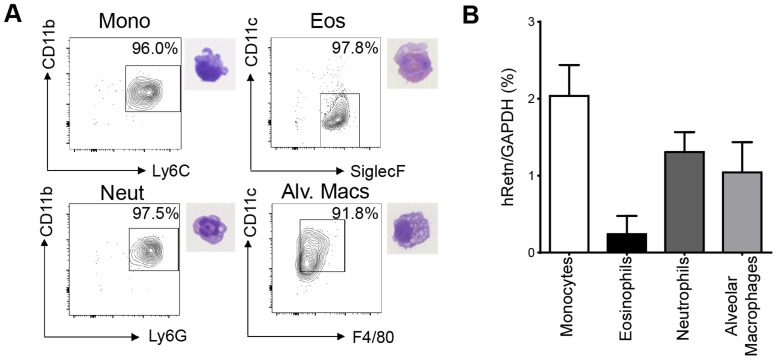
Monocytes, neutrophils and alveolar macrophages express hResistin in *Nb*-infected lungs. (A) Gating strategy for sorted lung cells from *Nb*-infected mice for CD11b^+^Ly6C^+^ monocytes, SiglecF^+^CD11c^−^ eosinophils, CD11b^+^Ly6G^+^ neutrophils, and CD11c^+^F4/80^+^ alveolar macrophages and corresponding H&E stained cytospins. (B) Sorted cells were recovered for RNA and analyzed for h*Retn* by real-time PCR. Data (mean ± SEM, n = 3–4 per group) are representative of three separate experiments.

To study the function of human resistin in helminth infection, we utilized mice in which the human resistin gene along with its entire regulatory region was inserted using a bacterial artificial chromosome onto a murine resistin knockout background (h*Retn*Tg^+^). Previous characterization of these mice showed that they provide a useful model for studying the effects of human resistin, as both humans and h*Retn*Tg^+^ mice have comparable levels of circulating resistin [25], [26]. h*Retn*Tg^+^ mice were infected with the STH *Nippostrongylus brasiliensis* (*Nb*), a parasite of mice and rats that infects the lungs and small intestine. *Nb* colonization of the lung and small intestine resulted in increased human resistin expression at the mRNA and protein level, as measured by real-time PCR and ELISA of the infected tissue respectively (Fig. 1A, B). Using immunofluorescent (IF) staining of lung sections from naïve and infected h*Retn*Tg^+^ mice, we quantified the frequency of hResistin^+^ cells as a percentage of the total number of DAPI^+^ cells. Compared to naïve mice, we observed significant increases in hResistin^+^ cells in the lung as early as day 2 post-infection, a time point at which the parasites are present in the lung (Fig. 1C). IF staining for hResistin (green) and *Griffonia simplicifolia* lectin (GSL), a macrophage-binding lectin (red) [30], revealed that hResistin was predominantly expressed by GSL^+^ macrophages (Fig. 1D). To systematically examine the cell-types that express hResistin, we sorted the dissociated cells from the lungs of *Nb*-infected Tg^+^ mice followed by real-time PCR analysis for h*Retn* (Fig. 2). Sorted cells were analyzed for purity by flow cytometry and hematoxylin and eosin (H&E)-stained cytospins, revealing >90% purity (Fig. 2A). CD11c^+^F4/80^+^ macrophages were the cell-types of highest frequency in the lung that expressed h*Retn*. In addition to macrophages, monocytes and neutrophils but not eosinophils expressed detectable h*Retn* mRNA (Fig. 2B). To our knowledge, this is the first demonstration that human resistin expression occurs in helminth-infected tissues and suggests that, in addition to metabolic disorders and cancer, human resistin may have a critical function in helminth infection.

### Human resistin leads to exacerbated pulmonary inflammation during *Nb* Infection

**Figure 3:**
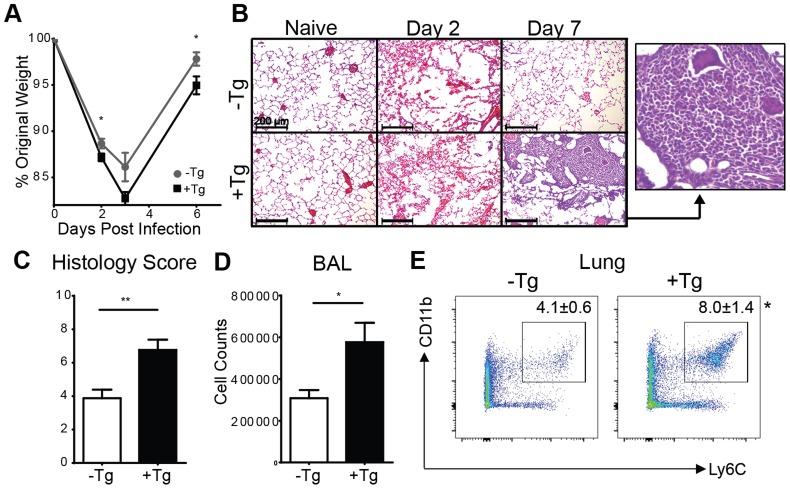
Expression of hResistin exacerbates lung inflammation. (A) h*Retn*Tg^−^ mice or h*Retn*Tg^+^ mice were infected with *Nb* and weight loss was measured as a percentage of original weight. (B-C) Lung sections from naïve or *Nb*-infected mice were stained with H&E (B, scale bar  = 200 µm) and pathology at day 7 post-infection was assessed by blind scoring (C). (D-E) At day 7 post infection, total BAL cells were quantified (D) and dissociated lung cells were recovered for flow cytometry analysis of Ly6C^+^ CD11b^+^ monocytes (E). Data (mean ± SEM, n = 4–6 per group) are representative of three separate experiments.

To examine the functional significance of the increased hResistin during *Nb* infection, h*Retn*Tg^+^ or littermate control h*Retn*Tg^−^ were infected with *Nb*. Compared to the control h*Retn*Tg^−^ mice, the h*Retn*Tg^+^ mice exhibited more severe weight loss beginning at day 2 post *Nb* infection (Fig. 3A). Histological examination of H&E-stained lung sections from h*Retn*Tg^−^ mice revealed that compared to naïve mice, *Nb* colonization of the lung resulted in hemorrhage and disruption of the lung alveolar architecture at day 2 post-infection, which was resolved by day 7 post infection, mirroring the weight recovery observed (Fig. 3B, upper panels). In contrast, h*Retn*Tg^+^ mice were unable to resolve inflammation by day 7 post-infection and instead exhibited increased leukocyte infiltration of the lung, characterized by the presence of large mononuclear cell-rich granulomas (Fig. 3B, lower panels and inset). The exacerbated lung inflammation in the h*Retn*Tg^+^ mice was confirmed by blind histology scoring (Fig. 3C), and was associated with significantly increased numbers of cells from bronchio-alveolar lavage (BAL) (Fig. 3D). To characterize the leukocyte infiltrates observed in the infected lungs, the lung tissue from h*Retn*Tg^−^ or h*Retn*Tg^+^ mice was dissociated and analyzed by flow cytometry. While there were no significant differences in eosinophils, neutrophils, CD4^+^ T cells or alveolar macrophages (S1A Fig.), we observed a significant increase in CD11b^+^Ly6C^+^ monocytes, suggesting that the granulomas observed in the lungs might consist of inflammatory monocytes (Fig. 3E). In conclusion, these results demonstrate a unique and specific effect for human resistin in promoting *Nb* infection-induced inflammation and inflammatory monocytes in the lung.

### Th2 cytokines are not altered by hResistin

Previous studies have shown that Th2 cytokines are critical in dampening the acute lung inflammation caused by *Nb* infection [8]. Additionally, studies from our group and others have shown that murine RELMα, which shares sequence identity and expression pattern with human resistin, suppresses Th2 cytokine responses in the lung [15], [16]. We therefore hypothesized that the exacerbated lung inflammation observed in the h*Retn*Tg^+^ mice was associated with reduced expression of Th2 cytokines or dysregulated RELMα expression. However, real-time PCR analysis of naïve or *Nb*-infected lung tissue from h*Retn*Tg^+^ or h*Retn*Tg^−^ mice revealed equivalent induction of RELMα and Th2 cytokines IL-4, IL-5 and IL-13 in both groups of mice (S1B Fig.). These data suggest that human resistin mediates exacerbated lung inflammation through a mechanism that is independent of Th2 cytokines and RELMα.

### Genome-wide transcriptional profiling reveals that global immune response pathways are triggered by hResistin

**Figure 4:**
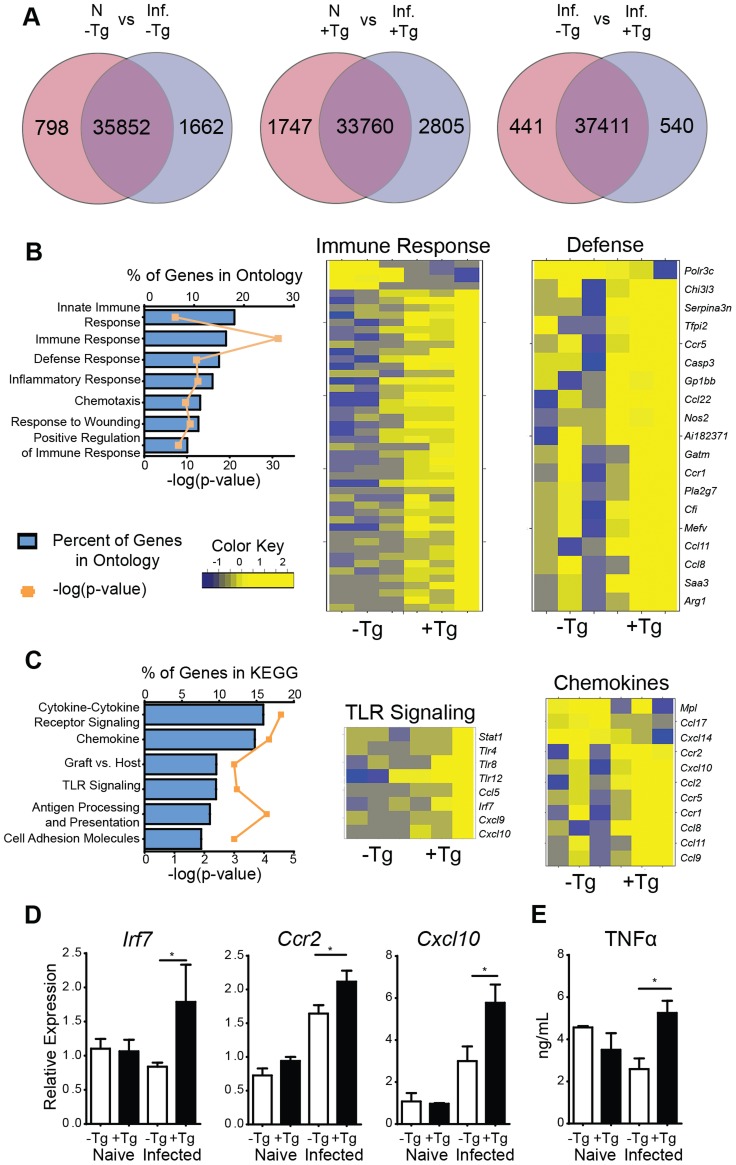
hResistin promotes type 1 inflammation, TLR signaling and chemokines. (A) Venn diagrams demonstrate infection-induced genes in h*Retn*Tg^−^ and h*Retn*Tg^+^ mice. (B-C) Enriched biological pathways induced by *hRetn* were identified by (B) Gene Ontology and (C) KEGG annotations and represented as a graph and heatmap. (D) h*Retn*-induced TLR signaling and chemokine genes were validated by real-time PCR analysis of lung tissue. (E) Serum TNFα was quantified by ELISA. Data (mean ± SEM, n = 3–4 per group) are representative of two separate experiments.

For an unbiased approach to identify gene expression pathways that are associated with hResistin-mediated inflammation in the lung, RNAseq analysis was performed on naïve or day 7 *Nb-*infected lung tissue from h*Retn*Tg^+^ or h*Retn*Tg^−^ mice. *Nb* infection resulted in global gene expression changes with 1662 genes upregulated by infection in the h*Retn*Tg^−^ mice, and a total of 2460 differentially expressed genes (DEG) (Fig. 4A, left). Interestingly, infection of h*Retn*Tg^+^ mice resulted in a greater number of genes upregulated (2805) with almost double the number of DEGs (4552) as h*Retn*Tg^−^ mice (Fig. 4A, middle). This suggests that hResistin has a significant impact on infection-induced gene expression changes in the lung. To assess infection-induced genes that were differentially expressed in response to hResistin, we compared *Nb*-infected h*Retn*Tg^−^ with h*Retn*Tg^+^ mice (Fig. 4A, right). We identified 540 genes upregulated in the h*Retn*Tg^+^ group and 441 genes downregulated for a total of 981 DEG. Gene set enrichment analysis revealed that the most significantly different gene ontology (GO) categories included the immune response, followed by the inflammatory response and defense response (Fig. 4B). Heatmap analysis revealed that almost all the GO-defined immune response genes and defense genes were upregulated in the h*Retn*Tg^+^ group, suggesting a potent immune-stimulatory function for human resistin. For example, the gene *Tnfrsf1b*, a receptor for the proinflammatory cytokine TNFα was highly upregulated in the h*Retn*Tg^+^ group. KEGG pathways were used to further specify the differentially expressed categories based on molecular interactions and functions (Fig. 4C). Specifically, two of the most upregulated pathways were chemokine signaling and TLR signaling. The chemokine signaling genes included *Ccr2* and *Cxcl10*, which are known to contribute to monocyte recruitment [31], [32]. Differentially expressed TLR signaling genes included *Irf7* and *Tlr4*, a previously identified receptor for hResistin [29]. We performed real-time PCR analysis of the naïve or infected lung tissue to validate the increased expression of some of the genes involved in TLR and chemokine signaling and observed significant upregulation of *Irf7*, *Ccr2* and *Cxcl10* in infected h*Retn*Tg^+^ mice compared to h*Retn*Tg^−^ mice (Fig. 4D). Additionally, we observed elevated TNFα in the serum of infected h*Retn*Tg^+^ mice (Fig. 4E), suggesting that hResistin promoted inflammatory cytokines systemically. In contrast, the pathways that were downregulated by hResistin were not associated with the immune response (S1 Table). Instead, we observed the downregulation of metabolic pathways such as arachidonic acid metabolism and oxidative phosphorylation. Both of these metabolic pathways have been associated with Th2 cytokine-activated alternatively activated macrophages [33], [34], which are protective in metabolic disorders such as insulin resistance or regulation of acute inflammation by Th2 cytokines [8], [35]. The downregulation of these protective pathways by hResistin are consistent with the exacerbated inflammation observed in h*Retn*Tg^+^ mice. Taken together, genome-wide transcriptional profiling and computational analysis suggest that hResistin initiates global gene expression changes in the *Nb*-infected lung tissue that contribute to increased TLR signaling and chemokines. These in turn promote a proinflammatory cytokine environment and exacerbated lung inflammation.

### hResistin recruits monocytes and promotes inflammatory gene expression *in vivo*

**Figure 5:**
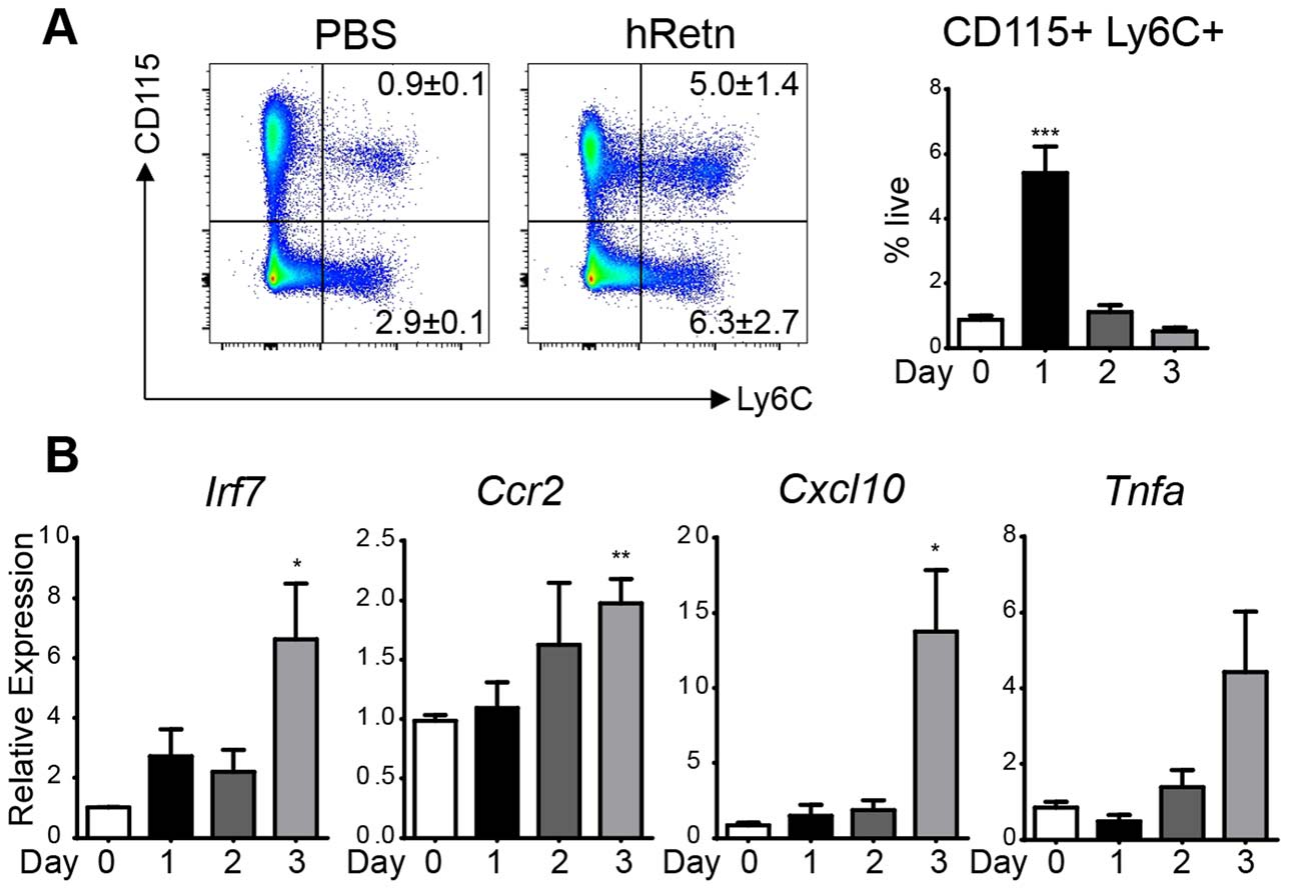
hResistin recruits inflammatory monocytes. (A-B) Naïve BL/6 mice were treated with recombinant human resistin (500 ng) followed by flow cytometry analysis of CD115^+^ Ly6C^+^ inflammatory monocytes (A) and real-time PCR analysis of PEC RNA (B). Data (mean ± SEM, n = 3 per group) are representative of two separate experiments.

Given that *Nb* infection of h*Retn*Tg^+^ mice was associated with increased expression of monocyte attractant chemokines and monocyte infiltration of the lung, we hypothesized that human resistin directly stimulated the production of chemokines leading to recruitment of monocytes and the establishment of a proinflammatory cytokine environment. To test this *in vivo*, we treated naïve mice with PBS or recombinant human resistin intraperitoneally (i.p.) followed by examination of cell recruitment to the peritoneal cavity and gene expression analysis. Recombinant human resistin treatment led to the rapid and brief recruitment of Ly6C^+^CD115^+^ inflammatory monocytes at day 1 post-injection, followed by a return to the monocyte frequency observed in PBS-treated mice at day 2 and 3 post-injection (Fig. 5A). This recruitment of leukocytes is specific for inflammatory monocytes, as no other cell population was increased at day 1 post infection (S2 Table). In contrast, real-time PCR analysis revealed a gradual significant increase in genes associated with TLR-signaling and chemokines that occurred after the peak in monocyte recruitment, culminating at day 3 post injection (Fig. 5B). To test that the effects of hResistin were specific and not caused by potential LPS contamination of the recombinant protein, we performed a limulus test to measure endotoxin levels in the recombinant protein. The endotoxin levels in the concentration of hResistin used were below the limit of detection of the assay. However, as a more appropriate control, we injected mice with LPS at the limit of detection of 1.5pg/mL. In comparison to LPS treatment, hResistin induced monocyte recruitment and proinflammatory gene expression (S2 Fig.), suggesting that hResistin effects were independent of LPS. Overall, this data corroborates the timing of *Nb* infection, as we see an increase in hResistin expression at day 3 post-infection (see Fig. 1A), with a subsequent increase in proinflammatory cytokines by day 7 post-infection (see Fig. 4).

### Monocytes and neutrophils are the main cellular targets of hResistin during *Nb* infection

**Figure 6:**
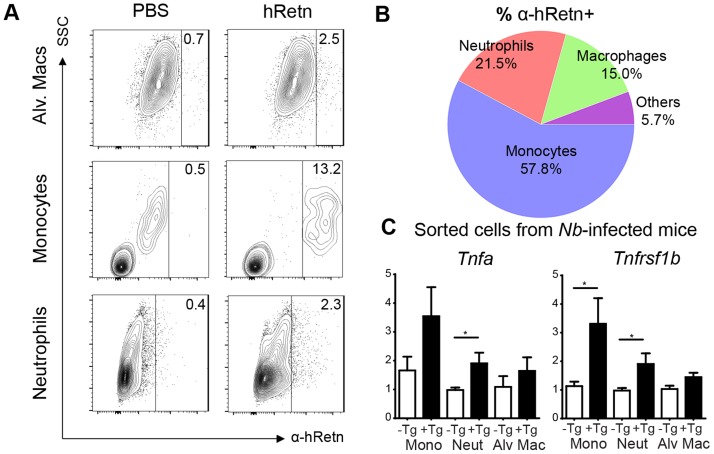
Monocytes and neutrophils are the main cellular targets of hResistin during *Nb* infection. (A-B) Lung cells from *Nb*-infected mice were incubated with recombinant hResistin followed by detection with α-hRetn to determine which cells can bind hResistin compared with control PBS (A). Surface expression of the hResistin-bound cells identified monocytes as the main cell-type that bound hResistin (B). (C) Sorted lung cells from *Nb*-infected h*Retn*Tg^−^ and h*Retn*Tg^+^ mice were analyzed for proinflammatory gene expression by real-time PCR. Data (mean ± SEM, n = 3–4 per group) are representative of two separate experiments.

In both *Nb* infection and treatment with recombinant hResistin, we observed increased monocyte responses, however, whether monocytes are the direct cellular target of hResistin in *Nb* infection is unclear. To examine this, we performed a resistin binding assay on dissociated lung cells from *Nb*-infected mice according to previously established methodologies from the lab [15]. Cells were incubated with recombinant hResistin or PBS, followed by detection with biotinylated anti-hResistin antibody. Compared to control PBS, hResistin preferentially bound lung monocytes with a distinct population of hResistin-bound monocytes (Fig. 6A, 13.2%). Further characterization of the hResistin-bound cells confirmed that monocytes constituted the highest proportion of resistin-bound cells (57.8%) followed by neutrophils (21.5%) and alveolar macrophages (15%) (Fig. 6B). The other cell-types examined, including eosinophils (SiglecF^+^CD11c^−^), T Cells (CD3^+^), dendritic cells (MHCII^hi^ CD11c^+^) and interstitial macrophages (MHCII^mid^ CD11b^+^), did not exhibit significant binding to hResistin, confirming that the hResistin binding assay was specific.

To assess the functional consequence of hResistin binding for expression of proinflammatory cytokines, we performed cell sorting of the lungs of *Nb-*infected h*Retn*Tg^+^ and h*Retn*Tg^−^ mice for monocytes (CD11b^+^ Ly6C^+^), neutrophils (CD11b^+^ Ly6G^+^) and alveolar macrophages (CD11c^+^ F4/80^+^) (Fig. 6C). Real-time PCR analysis for inflammatory genes *Tnfa* and *Tnfrsf1b* revealed that monocytes from the *hRetn*Tg^+^ mice exhibited the highest expression of proinflammatory genes, followed by neutrophils. In contrast, alveolar macrophages did not exhibit hResistin-mediated increases in proinflammatory gene expression. Together, these data implicate inflammatory monocytes and neutrophils as the cellular targets of hResistin-mediated expression of proinflammatory genes.

### h*Retn*Tg^+^ mice exhibit increased *Nb* egg and worm burdens

**Figure 7:**
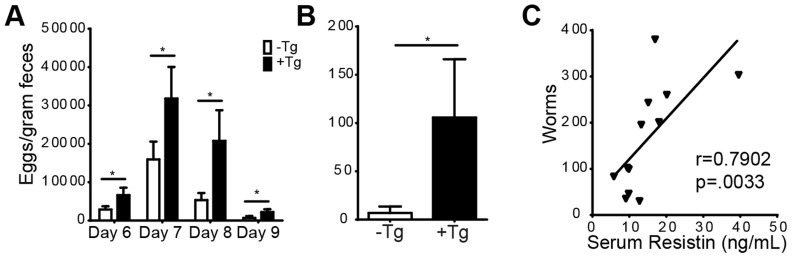
h*Retn*Tg^+^ mice are more susceptible to *Nb* infection. (A-B) h*Retn*Tg^+^ mice (black bars) and control h*Retn*Tg^−^ mice (white bars) were infected with 500 L3 *Nb* worms followed by parasitology analysis by fecal egg counts (A) or adult parasite numbers in the intestine at day 9 (B). Data (mean ± SEM, n = 4–6 per group) are representative of three separate experiments. (C) Serum hResistin concentration in h*Retn*Tg^+^ mice is positively correlated with *Nb* worms at day 7 post-infection (n = 12).

In human studies, a type 1 skewed immune response during helminth infection has been correlated with increased susceptibility to gastrointestinal nematode infection [36]. Additionally, mice deficient in TLR-4 are highly resistant to the gastrointestinal nematode, *Trichuris muris* [13]. We hypothesized that the type 1 proinflammatory environment and the increased TLR-4 signaling mediated by human resistin might therefore impair intestinal immunity to *Nb*. To this end, we monitored the time course of *Nb* colonization in the small intestine of h*Retn*Tg^+^ and h*Retn*Tg^−^ mice by assessing the parasite egg burdens in the feces followed by sacrifice of the mice at day 9 post-infection. Throughout the course of infection, h*Retn*Tg^+^ mice exhibited more than two-fold increases in parasite egg burdens (Fig. 7A). At day 9 post-infection, the majority of the h*Retn*Tg^−^ mice had expelled the *Nb* worms whereas the h*Retn*Tg^+^ mice had an average of 100 worms in the small intestine (Fig. 7B). In addition to infection-induced resistin expression in the small intestine (see Fig. 1), we examined if systemic hResistin expression could be predictive of higher worm burden. Analysis of serum human resistin levels in infected h*Retn*Tg^+^ mice from 3 combined experiments and a total of 12 mice revealed a significant and positive correlation between systemic human resistin expression and intestinal parasite burden (Fig. 7C). Overall, these data suggest that *Nb* parasites induce hResistin infection locally and systemically, adversely affecting parasite expulsion.

### Murine resistin does not affect *Nb*-induced inflammation or parasite burden

Given that human resistin caused dramatic effects in the immune response to *Nb*, we investigated if murine resistin mediated similar inflammatory effects. *Nb* infection of wild-type (WT) C57BL/6 mice did not induce murine resistin in the infected tissue, and we observed no difference in weight loss or parasite burdens between WT and m*Retn* ^−/−^ mice (S3 Fig.). It is likely that the difference in expression pattern between murine resistin, which is expressed in adipose tissue, and human resistin, which is expressed by monocytes/macrophages, accounts for the functional differences between murine and human resistin during helminth infection. Taken together, we have identified a unique function for human resistin in promoting type 1 inflammation and impeding parasite clearance during *Nb* infection.

### Resistin is upregulated in human soil-transmitted helminth infection and is positively correlated with proinflammatory cytokines

**Figure 8:**
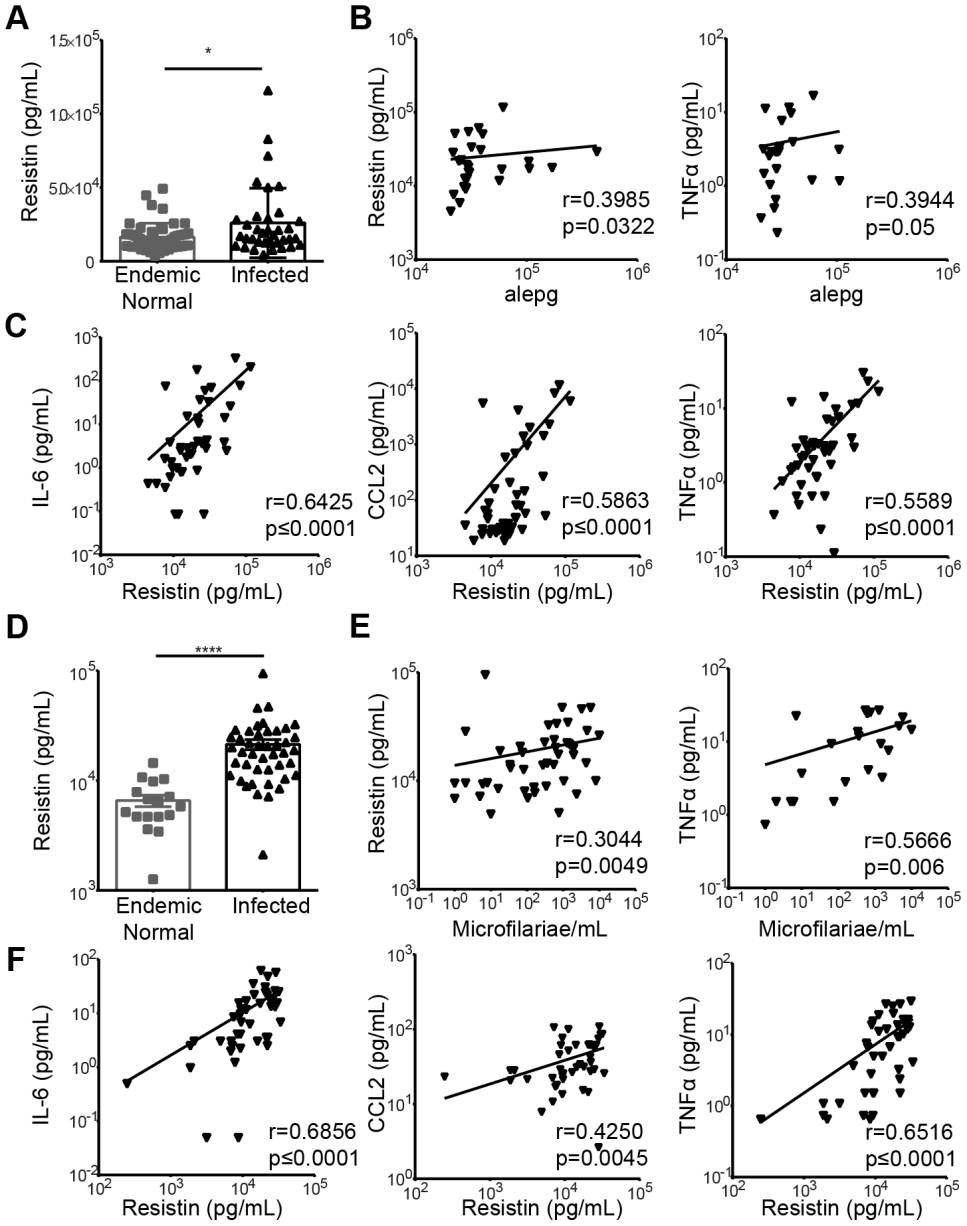
Expression of resistin in human patients infected with STH or filarial nematodes. (A) Serum resistin from uninfected (n = 51) or STH-infected children (n = 49) was measured. (B) The fecal egg burdens in infected children was quantified as *A. lumbricoides* eggs per gram feces (alepg) and plotted against serum resistin and TNFα. (C) Proinflammatory cytokines were positively correlated with resistin in the serum of STH-infected children. (D) Serum from uninfected endemic normal individuals (n = 17) or patients infected with *Wuchereria bancrofti* (n = 44) was analyzed by ELISA for resistin. (E) In infected individuals, a positive correlation was observed between the circulating microfilariae and resistin or TNFα. (F) Proinflammatory cytokines were positively correlated with resistin levels in the serum of infected patients.

STH are the most widespread helminth infection in humans, affecting an estimated 1 billion people worldwide. In many of these cases, environmental and economic factors promote high rates of co-infection with various parasites and reinfections by STH [37]. As *Nb* is an STH, we sought to investigate the clinical significance of our findings in the h*Retn*Tg^+^ mouse model. We compared serum collected from STH-infected school children resident in Ecuador (age 5–15, average 9 years) to uninfected, endemic normal school children from the same cohort [38]. The cohort included 49 infected and 51 uninfected school children. Within the infected cohort, all were infected with *A. lumbricoides*, many were infected with *T. trichiura*, and only a few were infected with *A. duodenale*, showing that *A. lumbricoides* was the most prevalent STH in that region [38]. Consistent with the h*Retn*Tg^+^ mouse model, infected individuals exhibited significantly increased expression of resistin compared to uninfected children (Fig. 8A). Given our previous observation that human resistin initiated a proinflammatory cytokine environment and impaired parasite expulsion following *Nb* infection, we tested if serum resistin expression was predictive of increased parasite burden or proinflammatory cytokine expression in the infected school children. We observed a significant and positive correlation between serum resistin expression and the *A. lumbricoides* egg burden (alepg) in the feces (Fig. 8B, left). Next, we performed luminex assays to measure the concentration of multiple cytokines in the serum. We chose proinflammatory cytokines (e.g. TNFα, CCL2, IL-6), which were initially identified in the h*Retn*Tg^+^ mouse model, and other factors that have been associated with helminth-associated morbidity for a total of 11 analytes. We observed that elevated TNFα serum levels were associated with increased parasite egg burdens in the feces (Fig. 8B, right). Given our transgenic mouse studies showing that resistin directly promotes TNFα expression, we hypothesized that helminth-induced resistin expression in the infected children promoted a proinflammatory cytokine environment that was responsible for impaired parasite clearance. We tested associations between serum resistin and proinflammatory cytokines, and observed a significant positive correlation between resistin and IL-6, CCL2 and TNFα (Fig. 8C), and other proinflammatory cytokines (Table 1). Interestingly, higher resistin levels also correlated with a higher concentration of wound healing factors, such as Chi3l1 and VEGF (Table 1), reflecting the RNAseq data (see Fig. 4). Overall, these results support our findings from the h*Retn*Tg^+^ mouse model, and confirm that our mouse model can be appropriately applied to human helminth infections.

**Table 1:** Many inflammatory factors are positively correlated with resistin in humans infected with soil-transmitted helminths or filarial nematodes.

	STH Infection		Filarial Nematode Infection	
	Spearman rho	P-value	Spearman rho	P-value
**Chi3l1**	***0.4825***	***0.0005***	0.0302	0.8475
**VEGF**	***0.4184***	***0.0031***	***0.3751***	***0.0132***
**IL-1α**	***0.4034***	***0.0120***	0.2439	0.1196
**IFNγR1**	***0.2699***	***0.0636***	***0.5809***	***≤0.0001***
**IFNγ**	0.3913	0.1491	0.1383	0.3763
**IL-6Rα**	0.0953	0.5193	***0.4369***	***0.0034***
**VCAM1**	0.0670	0.6510	0.2497	0.1108
**Leptin**	−0.0048	0.9740	0.1640	0.2933

### Resistin expression is elevated in human filarial nematode infection

Given that we have previously observed the induction of RELM proteins in various helminth infections of mice [17], [39], [40], we hypothesized that human resistin expression may be an indicator of multiple helminth infections. After STH infections, filariasis is the second most prevalent nematode infection, affecting an estimated 120 million people worldwide [1]. We examined serum samples collected from 44 filarial-infected individuals and 17 non-infected endemic normal individuals from the *Wuchereria bancrofti*-endemic island of Mauke in the south Pacific, where levels of circulating microfilariae were previously quantified in the blood [41], [42]. We observed that circulating resistin levels were increased in infected microfilaremic individuals by almost three-fold compared to the non-infected endemic normal population (average 6749 pg/mL in endemic normal versus 21361 pg/mL in infected individuals) (Fig. 8D). Microfilarial counts were also positively associated with resistin and TNFα (Fig. 8E). Finally, elevated resistin was associated with higher concentration of IL-6, CCL2 and TNFα (Fig. 8F) and inflammatory factors (Table 1), suggesting that the resistin/proinflammatory cytokine immune axis we identified in mice is also present in human nematode infections. Protective immunity to helminths is typically associated with type 2 cytokine responses [43], therefore we investigated if resistin expression was inversely correlated with parasite-specific type 2 immune responses in the microfilarial-positive patients. There was no significant correlation between resistin expression and parasite-specific antibody titers of the Th2-associated isotype IgG4, nor did we observe significant correlations with IL-5 or IL-10 cytokine production (S3 Table). This suggests that the downstream effects of resistin are independent of type 2 cytokines, and instead involve proinflammatory cytokine induction. Taken together, these human studies support a model where resistin is an innate response to multiple helminths that is correlated with elevated inflammatory cytokines and impaired parasite clearance.

## Discussion

Some of the earliest studies of RELM proteins identified these as potently induced genes in mouse helminth infection models [39]. Optimal expression of RELMα and RELMβ in helminth infection is dependent on Th2 cytokines [39], [44]. Recent studies, however, have reported that other stimuli, such as bacterial infections and presumably LPS, can induce RELMα and RELMβ gene expression [45], [46]. Similarly, human resistin expression is induced in a variety of inflammatory settings including endotoxemia, metabolic disorders and cancer [25], [27], [47]. However, the function of human resistin in infection has not been defined. Here, we combined studies utilizing a transgenic mouse model and human data to demonstrate that human resistin is induced following both STH and filarial nematode infection, where it promotes a proinflammatory cytokine environment. In the mouse model, the effects of resistin were detrimental to the host, and data from both the mouse and human studies demonstrated a positive correlation between resistin, proinflammatory cytokines and parasite burdens.

Previous studies showed that resistin mRNA was upregulated in macrophages from filarial-infected patients following exposure to filarial antigen [48], [49]. In h*Retn*Tg^+^ mice and the serum of STH and filarial nematode-infected individuals, we demonstrate that resistin protein is significantly elevated in the infected tissue and systemically in the circulation. Although many studies have shown that LPS can induce resistin expression both *in vitro* and *in vivo*, our data implicates resistin expression as a common innate response to multiple helminth infections. This suggests that hResistin is not only functionally relevant during type 1 inflammatory settings induced by bacterial antigens or metabolic disorders, but may have functional consequences during a Th2-biased helminth immune response.

Human resistin did not significantly alter the Th2 cytokine gene expression, or associated Th2 cell activation. Instead, we demonstrate through genome-wide transcriptional profiling and functional studies that resistin promoted the recruitment of inflammatory monocytes and the establishment of an innate proinflammatory cytokine environment, including increased expression of TLR-signaling associated genes and monocyte chemoattractants. This is consistent with previous *in vitro* studies utilizing human monocyte/macrophages [50]–[52]. In a co-culture system with endothelial and smooth muscle cells, resistin induced the expression of monocyte chemoattractants including CCL2, which led to increased monocyte migration [53]. Given that *Nb* infection of the lung causes dramatic injury and hemorrhage (see Fig. 2), it is likely that there is activation of endothelial and smooth muscle cells to produce monocyte chemoattractants. Indeed, lung monocyte infiltration in the h*Retn*Tg^+^ mice was associated with significantly elevated expression of CCL2, and serum resistin concentration in both STH and filarial nematode-infected individuals was positively correlated with CCL2. Interestingly, *in vivo* treatment of naïve mice with recombinant resistin led to rapid monocyte recruitment that preceded increased expression of monocyte chemoattractants. This suggests that monocytes are directly recruited by resistin and in turn begin to express chemokines as part of a positive feedback loop to increase inflammation.

Previous studies have identified several putative receptors for human resistin *in vitro*, including TLR-4 and CAP1, which can also induce NF-κB dependent induction of proinflammatory cytokines TNFα, IL-6 and IL-1β [29], [52], [54]. Our RNA sequencing studies revealed that human resistin promoted expression of TLR-4 (and other TLRs), but not CAP1. Additionally, direct injection of recombinant human resistin in naïve mice resulted in a marked increase in inflammatory genes downstream of TLR signaling, including IRF7. This suggests that resistin may act through TLR-4 and not CAP1 during *Nb* infection, and the importance of CAP1 in resistin-mediated effects may be more significant in metabolic disorders.

Altogether, our data suggest that resistin expression could have significant implications for the disease outcome of helminth-infected individuals. In humans, increased resistin expression could be predictive of impaired immunity to helminths or exacerbated inflammation following infection. Recent studies have shown that filarial nematode and STH-infected individuals demonstrate increased circulating microbial products [55], [56]. It is possible that this endotoxemic phenotype promotes resistin expression, which in turn acts to increase the proinflammatory cytokine environment. In filarial nematode infection, the establishment of chronic infections typically involves the downmodulation of innate and adaptive immune responses [57]. Therefore, it is somewhat surprising that there was a significant and positive correlation between microfilarial burdens, resistin and proinflammatory cytokines in the chronically-infected patients. It is possible that the resistin-mediated effects on proinflammatory cytokines operate independently of regulatory mechanisms such as regulatory T cells and IL-10 that also contribute to chronicity of infection. One debilitating consequence of filarial nematode infections is the development of lymphatic dysfunction, lymphedema and ultimately elephantiasis. This pathology has been associated with elevated TLR signaling [12], and resistin may initiate this by overcoming the suppressive mechanisms that typically occur in filarial infections. Although there were not sufficient filarial nematode-infected individuals with lymphatic pathology in our cohort to test this hypothesis, our data suggest that future studies examining resistin correlation with lymphatic pathology may be warranted. Additionally, our studies of the transgenic mouse model and STH-infected children have provided a useful framework for longitudinal studies to examine whether high resistin expression leads to an increased likelihood of infection with STH. In summary, our studies contribute to the growing evidence of the pleiotropic effects of human resistin in several human diseases, and demonstrate a critical function for this protein in helminth infection. We show that expression of human resistin in mice recruits inflammatory monocytes to the site of infection, promotes type 1 inflammation, and is correlated with increased STH and filarial nematode burden in humans. To our knowledge, this is the first report of the ability of the human resistin to impair immunity to helminths and promote inflammatory responses that may mediate pathology. Targeting resistin may therefore provide new approaches to host-directed treatment strategies for helminth infection and amelioration of the associated immune-mediated pathology.

## Materials and Methods

### Mice

Human resistin transgenic mice (h*Retn*Tg^+^) were generated as previously described by Mitchell Lazar [26]. Briefly, the human resistin gene, along with 21,300 bp upstream and 4,248 bp downstream of the human resistin start site, were engineered through a bacterial artificial chromosome. h*Retn*Tg^−^ mice were backcrossed onto WT C57BL/6 mice bred in house to generate mRetn^−/−^ mice. In the resistin binding assay, CX3CR1^GFP^PGRP^dsred^ mice were used as reporters for monocytes and neutrophils respectively [58]. Recombinant human resistin (Peprotech) was used for *in vivo* intraperitoneal injections (500 ng/mouse at days -1 and/or day -3). In some experiments, mice were also treated intratracheally with 500 ng hResistin. Cells from the peritoneal cavity were recovered at 24, 48, and 72 hours and analyzed by flow cytometry and real-time PCR. Recombinant hResistin was tested for endotoxin contamination using the Pierce *Limulus* Amebocyte Lysate assay (Thermo Scientific) according to manufacturer's instructions under sterile conditions. For controls, mice were injected i.p. with PBS or the limit of detection concentration of LPS (1.5pg/mL). All animals in the experiment were age-matched (6–10 week old) and gender-matched, and housed five per cage under ambient temperature with 12 hour light/dark cycle.

### Infection

*Nippostrongylus brasiliensis* (*Nb*) life cycle was maintained in Sprague-Dawley rats purchased from Harlan Laboratories. Mice were injected subcutaneously with 500 *Nb* third-stage larvae (L3) and sacrificed at days 2, 7 and 9 post-infection. Eggs in the feces of infected mice were counted using a McMaster counting chamber on days 6–9 following infection. Adult worms within the small intestine were enumerated after the entire small intestine of infected mice was cut longitudinally and incubated in PBS at 37°C for >1 hr to allow worms to migrate out of the tissue.

### Cytokine Quantification

For sandwich ELISA, Greiner 96-well medium bind plates were coated with primary antibody to cytokines (Peprotech) overnight at room temperature. After blocking the plates with 5% NCS in PBS for 1 hr, sera or tissue homogenates were added at various dilutions and incubated at room temperature for 2 hr. Detection of cytokines was done with biotinylated antibodies for 2 hr, followed by incubate with streptavidin-peroxidase (Jackson Immunobiology) for 30 min. The peroxidase substrate TMB (BD) was added followed by addition of 2N H_2_SO_4_ as a substrate stop, and the optical density (OD) was captured at 450 nm. Samples were compared to a serial-fold dilution of recombinant cytokine.

### Human Studies

Serum and feces were collected from uninfected (n = 51) or STH-infected school children (n = 49) and helminth eggs in the feces were quantified by a modified Kato Katz or formol-ether concentration protocol [38]. For filarial-infected individuals, blood was collected and circulating microfilariae were previously quantified through filtration of 1 mL of whole blood into a Nucleopore 3 µm polycarbonate filter as previously described [41]. The patient group consisted of *Wuchereria bancrofti*-infected patients (n = 44) from the Cook island of Mauke and endemic normal individuals (i.e. those with no history of filarial infection at time of collection, n = 17). Custom Luminex assay kits (analytes TNFα, Chi3l1, IL-6Rα, MCP2, Leptin, VCAM1, IL-6, VEGF, IFNγ, IL-1α, IFNγR1) from R&D Systems were run according to manufacturer's instructions and quantified on Luminex 200 (Luminex Corp.).

### Real-time PCR

Tissue recovered for RNA extraction was first incubated overnight in RNAlater (Qiagen) at 4°C. Tissue RNA was extracted with Trizol and RNA from cells was extracted on RNeasy columns (Qiagen). iScript Reverse Transcriptase (Biorad) was used for cDNA synthesis. Relative quantification of cDNA was measured by real-time PCR using the Biorad CFX Connect. Primer sequences for real-time PCR are: *Ccl2* F-TGGCTCAGCCAGATGCAGT, R-TTGGGATCATCTTGCTGGTG; *Cxcl10* F-GCCGTCATTTTCTGCCTCA, R-CGTCCTTGCGAGAGGGATC; *Ccr2* F-TCAACTTGGCCATCTCTGACC, R-AGACCCACTCATTTGCAGCAT; *Irf*7 F-CAGCGAGTGCTGTTTGGAGAC, R-AAGTTCGTACACCTTATGCGG. All other primers were purchased from Qiagen. Ensembl ID for each gene: *Irf7* (ENSMUSG00000025498), *Ccr2* (ENSMUSG00000049103), *Tnfa* (ENSMUSG00000024401), *Cxcl10* (ENSMUSG00000034855), *Tnfrsf1b* (ENSMUSG00000028599), *Retnla* (ENSMUSG00000061100), *Il-4* (ENSMUSG00000000869), *Il-5* (ENSMUSG00000036117), *Il-13* (ENSMUSG00000020383).

### RNA Sequencing

RNA was originally extracted using Trizol reagent, and further purified on RNeasy columns (Qiagen). A total of 3 µg of RNA was used in synthesis of RNA library for sequencing, with periodic analysis of RNA and cDNA quality on the 2100 BioAnalyzer (Agilent Technologies). cDNA libraries from whole lung RNA were made using NEB ultra-directional RNA library kit with multiplexing primers. Multiplexed samples were sequenced on Illumina 2000 at the University of California, Riverside Genomics Core. Once sequenced, samples had 10–30 million total reads. Upon indexing and alignment through TopHat, read counts and normalization was done with R Bioconductor. Differentially expressed genes (DEGs) were defined in our study as being infection-induced, with a 1.5x fold change (h*Retn*Tg^+^ infected vs h*Retn*Tg^−^ infected), false discovery rate ≤0.05, and a p-value ≤0.05. Raw data was normalized and analyzed using QuasR package on R Bioconductor. DEGs were then categorized by DAVID Functional Annotation Tools using Gene Ontology Enrichment Terms and KEGG Pathways. RNA-seq dataare available on GEO (accession number GSE60537).

### Histology

Lungs were inflated with 1 mL 1 part 4% PFA/30% sucrose and 2 parts OCT and stored overnight in 4% PFA at 4°C. After 24 hours, lungs were removed from 4% PFA and incubated another 24 hours in 30% sucrose. Lungs were then blocked in OCT and sectioned at 8 µm. H&E-stained lung sections were blindly scored on a 1–5 scale with 5 being the most severe score of pathology using criteria of leukocyte infiltration (1–5) and vascular inflammation/endothelial cell hyperplasia (1–5) for a total score out of 10. Scoring of the lung section was based on the following: 1 – absent, 2 – slight, 3 – moderate (covering up to 5% of total area), 4 – marked (>5% and <10% of total area), and 5 – severe (covering ≥10% of total area). For immunofluorescence staining, sections were incubated with rabbit anti-hResistin (1∶400, generated by Mitchell Lazar), biotinylated *Griffonia simplicifolia* lectin (1∶400, Vector Laboratories) overnight at 4°C. Sections were incubated with appropriate fluorochrome-conjugated secondary antibodies for 2 hours at 4°C and counterstained with DAPI. Sections were visualized under a Leica microscope (DM5500 B) and Volocity software (PerkinElmer) was used to quantify number of hResistin^+^ and DAPI^+^ cells.

### Flow Cytometry

Lung tissue was cut up, incubated in 30 µg/mL DNAse and 1 mg/mL collagenase for 30 minutes in a 37°C shaking incubator and passed through a 70 µm cell strainer to generate single cell suspensions. BAL cells were recovered through washing with 3 mL of ice cold PBS. Peritoneal exudate cells (PEC) were recovered in a total of 5 mL of ice cold PBS. For flow cytometry, lung, BAL and PEC were blocked with 0.6 µg Rat IgG and 0.6 µg αCD16/32 (2.4G2, 5′), stained for 25′ with antibodies for SiglecF (E50-2440), Ly6G (1A8), MHCII (M5/114.15.2) all from BD Biosciences; F4/80 (BM8), CD115 (AFS98), Ly6C (HK1.4), CD3 (17A2), CD11b (M1/70), CD11c (N418) all from eBioscience, Affymetrix; CD4 (RM4-5 from Invitrogen). Cells were then washed and analyzed on the LSRII (BD Bioscience), followed by data analysis using FlowJo v10 (Tree Star Inc.). Cells were sorted by flow cytometry using FACSAria (BD Bioscience), and re-analyzed for purity on the LSRII (BD Bioscience).

### hResistin Binding Assay

Dissociated lung cells were collected from *Nb* infected mice and washed with FACS buffer prior to 1 hour incubation with 0.5 µg recombinant hResistin (Peprotech) or PBS for control. Cells were washed 2X in FACS buffer, incubated with Fc block (αCD16/32, 15′), stained with biotinylated αhRetn (Peprotech, 30′), followed by detection with BV421-conjugated streptavidin (BD Biosciences, 30′).

### Statistical Analysis

All statistics were analyzed by Graphpad Prism using where appropriate the student's t-test (for normal distribution data), Mann-Whitney nonparametric test (for asymmetric distribution data), two-way ANOVA (for analysis of more than one experiment), or nonparametric spearman correlation (for correlation analysis). *, p≤0.05; **, p≤0.01; ***, p≤0.0001.

### Ethics Statement

All protocols for animal use and euthanasia were approved by the University of California Riverside Institutional Animal Care and Use Committee (https://or.ucr.edu/ori/committees/iacuc.aspx; protocol A-20120023B and A-20120024E), and were in accordance with National Institutes of Health guidelines. Animal studies are in accordance with the provisions established by the Animal Welfare Act and the Public Health Services (PHS) Policy on the Humane Care and Use of Laboratory Animals. For the human studies, protocols were approved by the Cook Islands government and the NIAID Institutional Review Board for the Cook Islands study, and by the Ethics Committees of the Universidad San Francisco de Quito and the Hospital Pedro Vicente Maldonado for the Ecuador study. Samples were collected following the informed written consent of the patient or parent of the school children. Multiplex cytokine assays and resistin ELISAs were performed at UCR with the approval of the University of California Riverside Institutional Review Board (HS-13-134 and HS-14-048).

## Supporting Information

S1 Fig **Analysis of BAL cells and Th2 immune responses in** ***Nb*** **-infected h** ***Retn*** **Tg^−^ and h** ***Retn*** **Tg^+^ mice.** (A-B) Flow cytometric analysis of BAL cell populations (A) and real-time PCR analysis of *Retnla* and Th2 cytokines normalized to *Gapdh* (B) were performed on lung tissue from naïve or day 7 *Nb*-infected mice. Alv. Mac, alveolar macrophages. Data (mean ± SEM, n = 3–6 per group) are representative of three separate experiments.
(TIF)

S2 Fig **Effects of recombinant hResistin are independent of potential LPS contamination.** Naïve mice were treated i.p. with 500 ng recombinant hResistin or 0.15 pg LPS. (A-B) PECs were recovered at day 3 and measured for monocytes by flow cytometry (A) or proinflammatory cytokines by real-time PCR (B). Data represent mean ± SEM (n = 4 per group).
(TIF)

S3 Fig **Expression and role of murine resistin in** ***Nb*** **-infected mice.** Control WT C57BL/6 and murine resistin knockout (KO) mice were infected with 500 L3 *Nb* worms and sacrificed at day 7 post infection. (A) Real-time PCR analysis of mResistin expression in C57BL/6 mice following *Nb* infection was performed. (B) Weight loss following infection was monitored. (C-D) Fecal egg burden (C) and adult *Nb* worms (D) were quantified at day 7 post-infection. Data (mean ± SEM, n = 4 per group) are representative of three separate experiments.
(TIF)

S1 Table **KEGG pathway analysis reveals gene sets downregulated by hResistin.**
(DOCX)

S2 Table **Cell composition in the peritoneal cavity following recombinant hResistin injection.**
(DOCX)

S3 Table **Type 2 immunity is not altered by resistin in filarial-infected individuals.**
(DOCX)
