# Supplementary figures and images for: Macrophage-Derived Human Resistin Is Induced in Multiple Helminth Infections and Promotes Inflammatory Monocytes and Increased Parasite Burden
Source: PLoS Pathog. 2015 Jan 8;11(1):e1004579. doi: 10.1371/journal.ppat.1004579 (PMC4287580; doi:10.1371/journal.ppat.1004579)

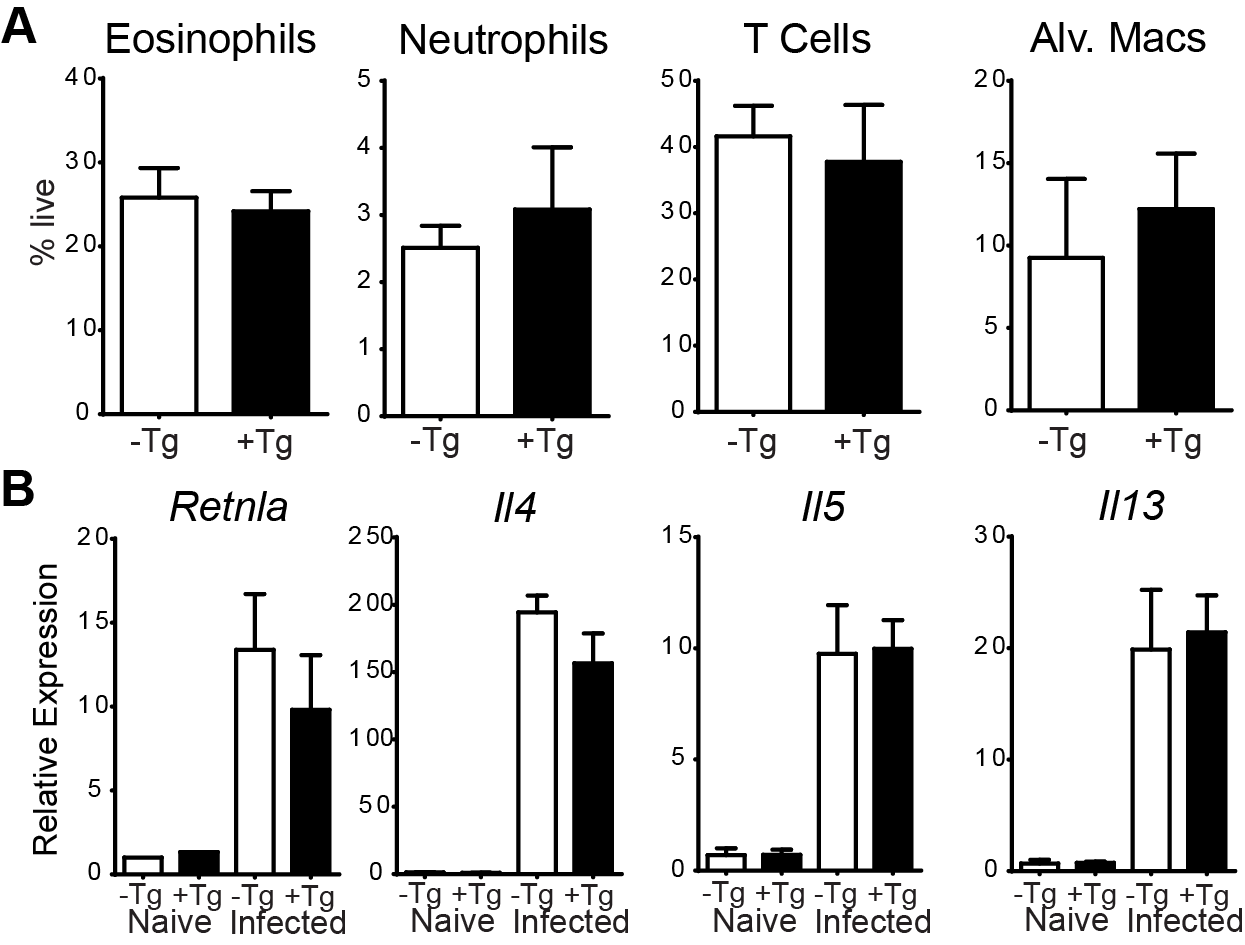

Supplement: S1 Fig — Analysis of BAL cells and Th2 immune responses in Nb -infected h Retn Tg− and h Retn Tg+ mice. (A-B) Flow cytometric analysis of BAL cell populations (A) and real-time PCR analysis of Retnla and Th2 cytokines normalized to Gapdh (B) were performed on lung tissue from naïve or day 7 Nb-infected mice. Alv. Mac, alveolar macrophages. Data (mean ± SEM, n = 3–6 per group) are representative of three separate experiments. (TIF) [file ppat.1004579.s001.tif]

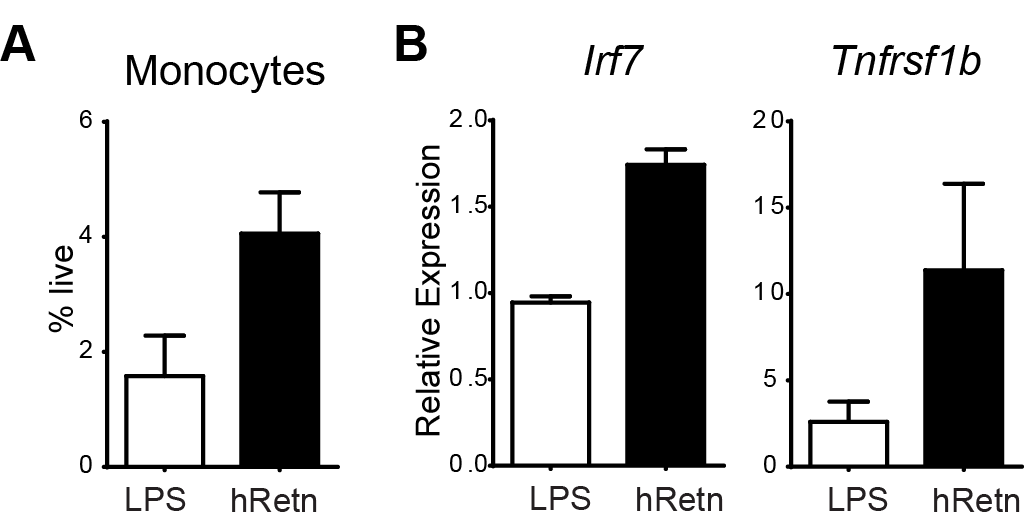

Supplement: S2 Fig — Effects of recombinant hResistin are independent of potential LPS contamination. Naïve mice were treated i.p. with 500 ng recombinant hResistin or 0.15 pg LPS. (A-B) PECs were recovered at day 3 and measured for monocytes by flow cytometry (A) or proinflammatory cytokines by real-time PCR (B). Data represent mean ± SEM (n = 4 per group). (TIF) [file ppat.1004579.s002.tif]

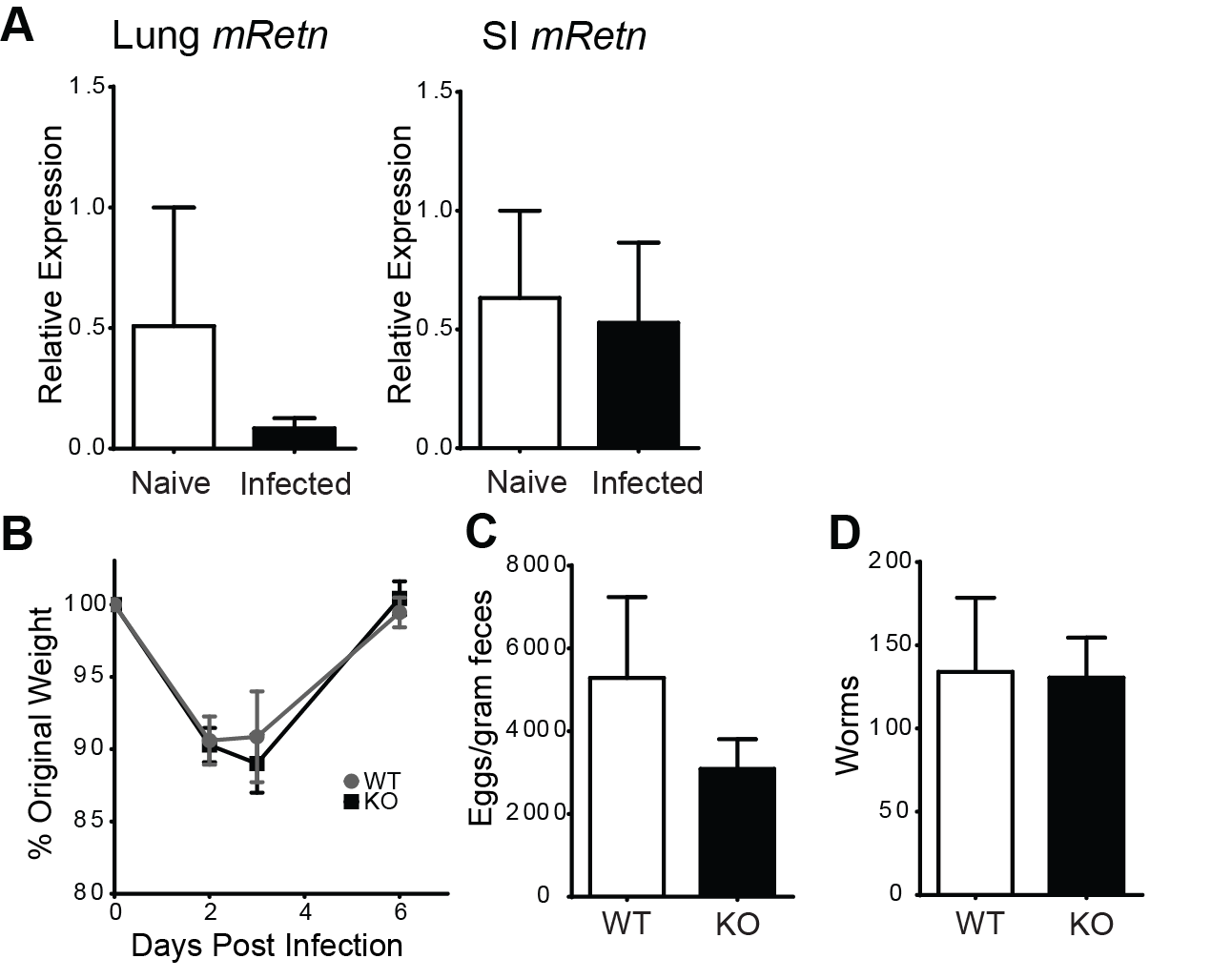

Supplement: S3 Fig — Expression and role of murine resistin in Nb -infected mice. Control WT C57BL/6 and murine resistin knockout (KO) mice were infected with 500 L3 Nb worms and sacrificed at day 7 post infection. (A) Real-time PCR analysis of mResistin expression in C57BL/6 mice following Nb infection was performed. (B) Weight loss following infection was monitored. (C-D) Fecal egg burden (C) and adult Nb worms (D) were quantified at day 7 post-infection. Data (mean ± SEM, n = 4 per group) are representative of three separate experiments. (TIF) [file ppat.1004579.s003.tif]
