# Supplementary material for: Macrophage-Derived Human Resistin Is Induced in Multiple Helminth Infections and Promotes Inflammatory Monocytes and Increased Parasite Burden
Source: PLoS Pathog. 2015 Jan 8;11(1):e1004579. doi: 10.1371/journal.ppat.1004579 (PMC4287580; doi:10.1371/journal.ppat.1004579)
Supplement: S1 Table — KEGG pathway analysis reveals gene sets downregulated by hResistin. (DOCX) [file ppat.1004579.s004.docx]

**Table S1: KEGG pathway analysis reveals gene sets downregulated by hResistin.**

| Term | P-value | Benjamini |
| --- | --- | --- |
| Ribosome | 3.2E-6 | 2.2E-4 |
| Cardiac Muscle Contraction | 1.2E-3 | 4.2E-2 |
| Drug Metabolism | 7.6E-3 | 1.5E-1 |
| Arachidonic Acid Metabolism | 1.1E-2 | 1.7E-1 |
| Oxidative Phosphorylation | 1.1E-2 | 1.4E-1 |
| Metabolism of Xenobiotics by Cytochrome p450 | 3.1E-2 | 3.1E-1 |
| RNA Polymerase | 3.2E-2 | 2.7E-1 |
| Retinol Metabolism | 3.4E-2 | 2.6E-1 |
| Huntington’s Disease | 4.2E-2 | 2.8E-1 |
| Parkinson’s Disease | 5.0E-2 | 3.1E-1 |
