# Supplementary material for: Macrophage-Derived Human Resistin Is Induced in Multiple Helminth Infections and Promotes Inflammatory Monocytes and Increased Parasite Burden
Source: PLoS Pathog. 2015 Jan 8;11(1):e1004579. doi: 10.1371/journal.ppat.1004579 (PMC4287580; doi:10.1371/journal.ppat.1004579)
Supplement: S2 Table — Cell composition in the peritoneal cavity following recombinant hResistin injection. (DOCX) [file ppat.1004579.s005.docx]

**Table S2: Cell composition in the peritoneal cavity following recombinant hResistin injection**

|  | PBS (%live ±SEM) | hResistin (% live±SEM) | p-value |
| --- | --- | --- | --- |
| Monocytes | 0.92±0.13 | 5.0±1.4 | 0.0006 |
| T Cells | 6.14±0.51 | 4.01±0.81 | 0.0916 |
| Eosinophils | 24.97±2.73 | 22.26±7.77 | 0.7586 |
| Neutrophils | 0.85±0.47 | 1.61±1.18 | 0.5797 |
| Macrophages | 28.70±9.22 | 40.65±9.85 | 0.4555 |
