# Supplementary material for: Macrophage-Derived Human Resistin Is Induced in Multiple Helminth Infections and Promotes Inflammatory Monocytes and Increased Parasite Burden
Source: PLoS Pathog. 2015 Jan 8;11(1):e1004579. doi: 10.1371/journal.ppat.1004579 (PMC4287580; doi:10.1371/journal.ppat.1004579)
Supplement: S3 Table — Type 2 immunity is not altered by resistin in filarial-infected individuals. (DOCX) [file ppat.1004579.s006.docx]

**Table S3: Type 2 immunity is not altered by resistin in filarial-infected individuals**

|  | Spearman rho | p-value |
| --- | --- | --- |
| Serum | | |
| IgG4 Mf | 0.1026 | 0.4782 |
| PBMC stimulated with Mf antigen | | |
| IL-10 | 0.038 | 0.802 |
| IL-5 | -0.099 | 0.511 |
| IFNγ | 0.279 | 0.061 |
